# Supplementary material for: Resolving multi-image spatial lipidomic responses to inhaled toxicants by machine learning
Source: Nat Commun. 2025 Mar 26;16:2954. doi: 10.1038/s41467-025-58135-4 (PMC11947182; doi:10.1038/s41467-025-58135-4)
Supplement: Supplementary file 1 — Supplementary Information [file 41467_2025_58135_MOESM1_ESM.pdf]

Supplemental Material for Resolving multi-image spatial lipidomic responses to inhaled toxicants by machine learning

Nathanial C. Stevens[1], Tong Shen[1], Joshua Martinez[2], Veneese J. B. Evans[2], Morgan C. Domanico[2], Elizabeth K. Neumann[3], Laura S. Van Winkle[2,4] and Oliver Fiehn[1]

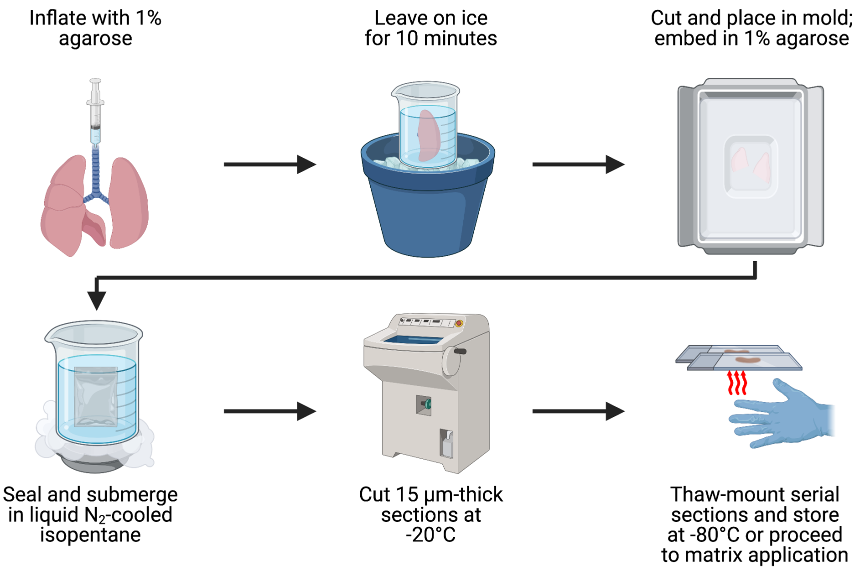

Supplementary Figure 1. Sample preparation protocol used to prepare lung slices for matrix application and MALDI TOF-MS analysis. Lung lobes were cut along the axial plane prior to embedding with agarose to obtain 15 μm lung cross-sections for analysis. Created in BioRender. Stevens, N. (2025) https://BioRender.com/r40a000.

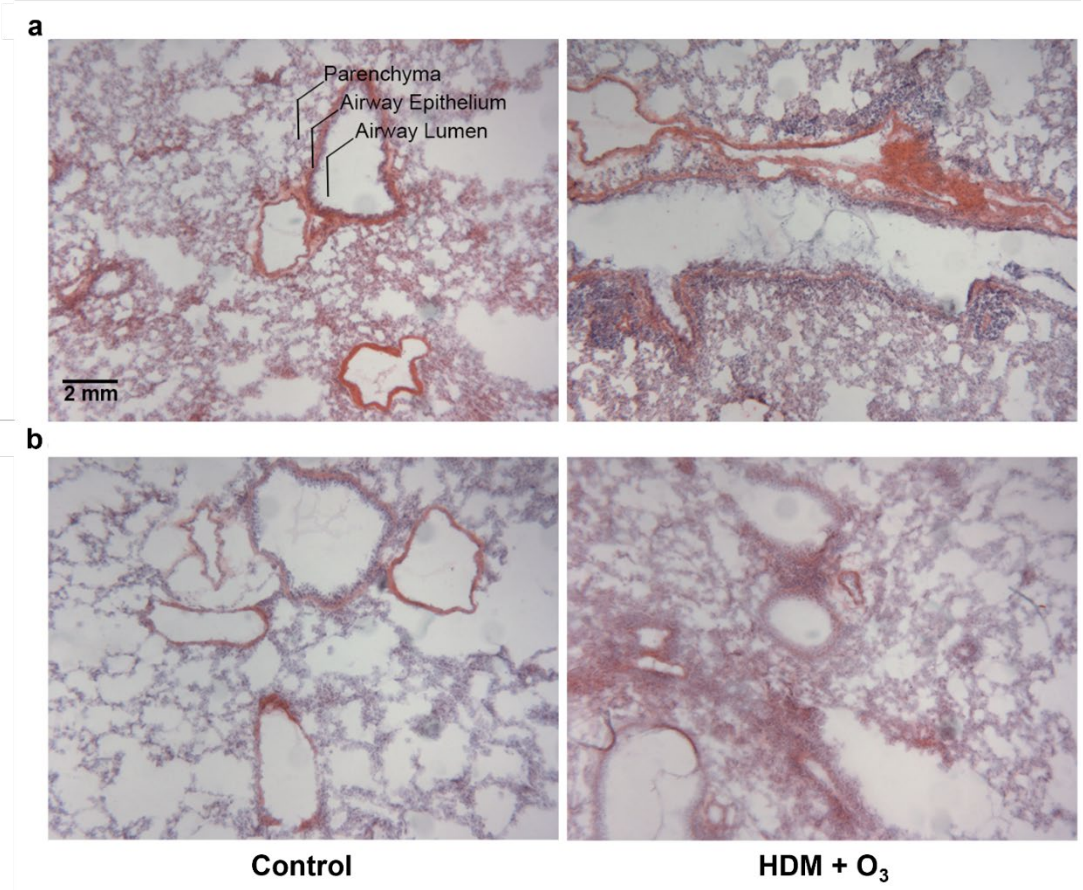

Supplementary Figure 2. Validation of selected regions using serial H&E sections. Representative H&E sections from control and HDM + O_3 exposed (**a**) male and (**b**) female mice. The airway epithelium, airway lumen, and lung parenchyma are labeled and confirmed the regions isolated in Figure 3. 4x magnification, Bar = 2 mm. Representative images were selected from three independent sections for each condition (n=3/group, total n=12).

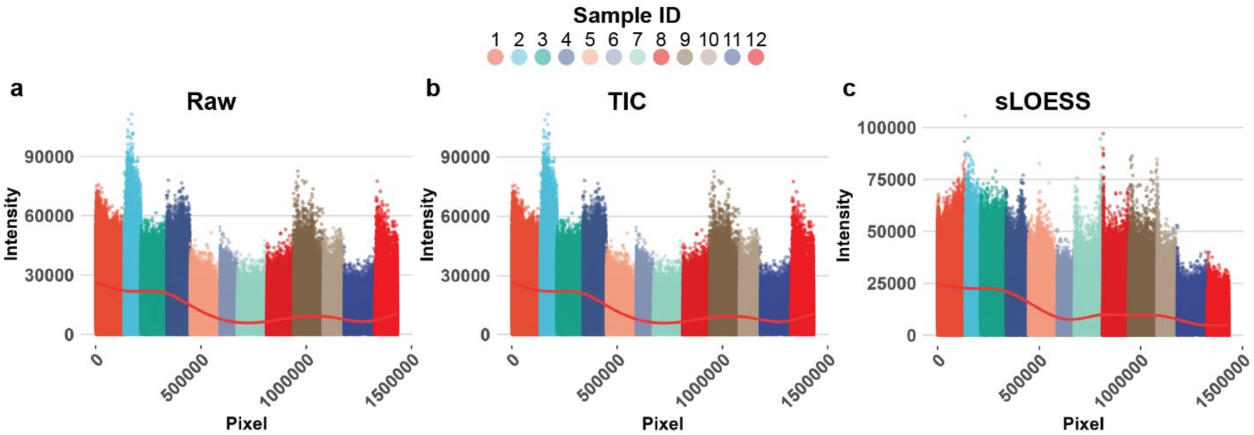

Supplementary Figure 3. Comparison of TIC and sparse LOESS normalization of positive ionization mode MSI data. Total ion current scatterplots of signal intensity vs. pixel number according to acquisition order in positive ionization mode for **a)** raw, **b)** TIC, and **c)** sparse LOESS normalized data. TIC values correspond to the sum intensity of all annotated compounds for each pixel. Sample ID reflects the identity of individual imaging runs in order of data acquisition. Source data for panels a-c are provided as Source Data files.

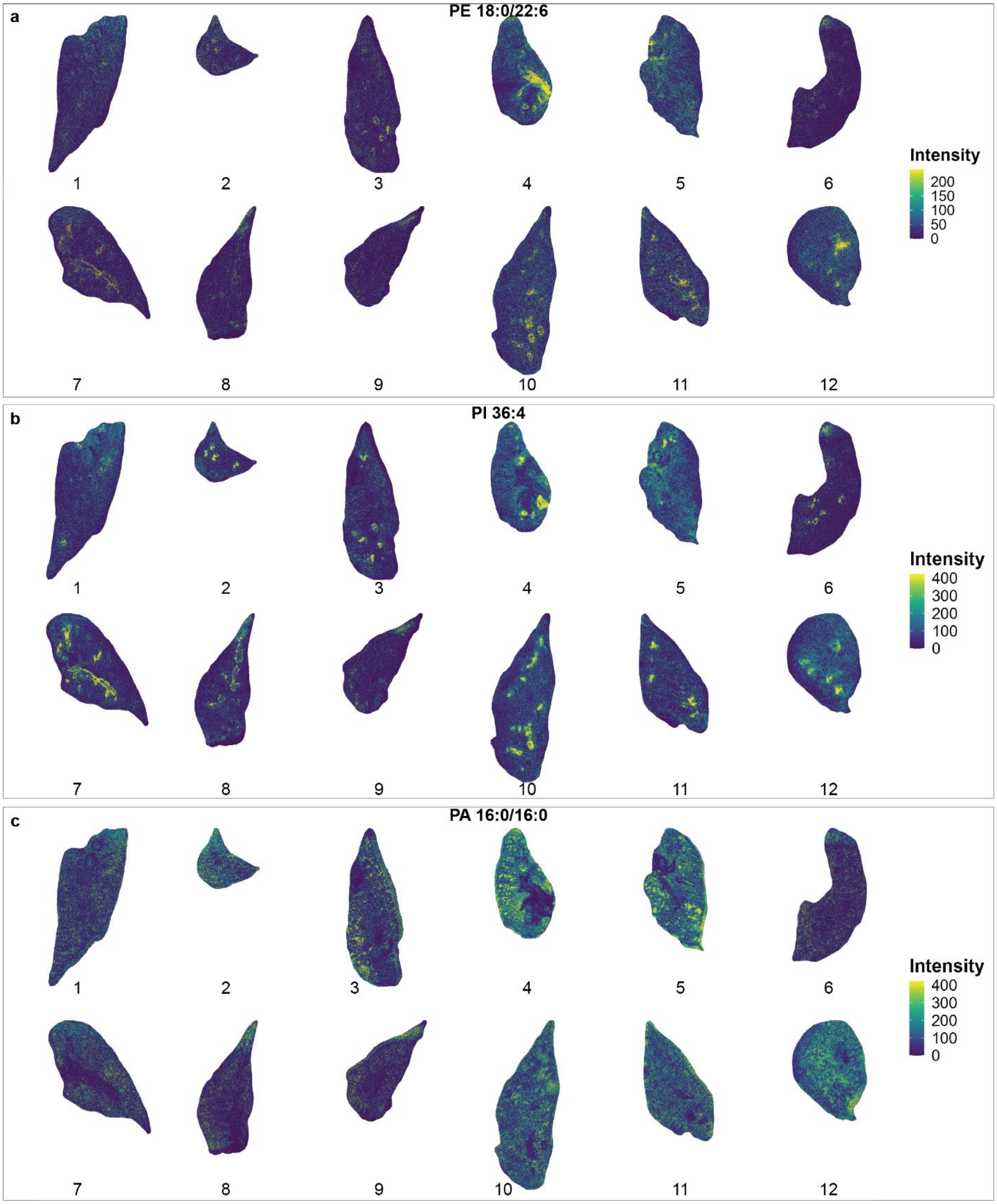

Supplementary Figure 4. Spatial distribution of individual phospholipid species in sparse LOESS-normalized ion images. Ion images representing pixel intensities of annotated phospholipids in negative mode, including **a)** PE 18:0/22:6, **b)** PI 36:4, and **c)** PA 16:0/16:0. Intensity distributions are included for each individual lung lobe acquired by negative mode and numbered in order of data acquisition. Distinct acyl chains were not annotated for PI 36:4 based on LC-MS/MS validation and reference matching.

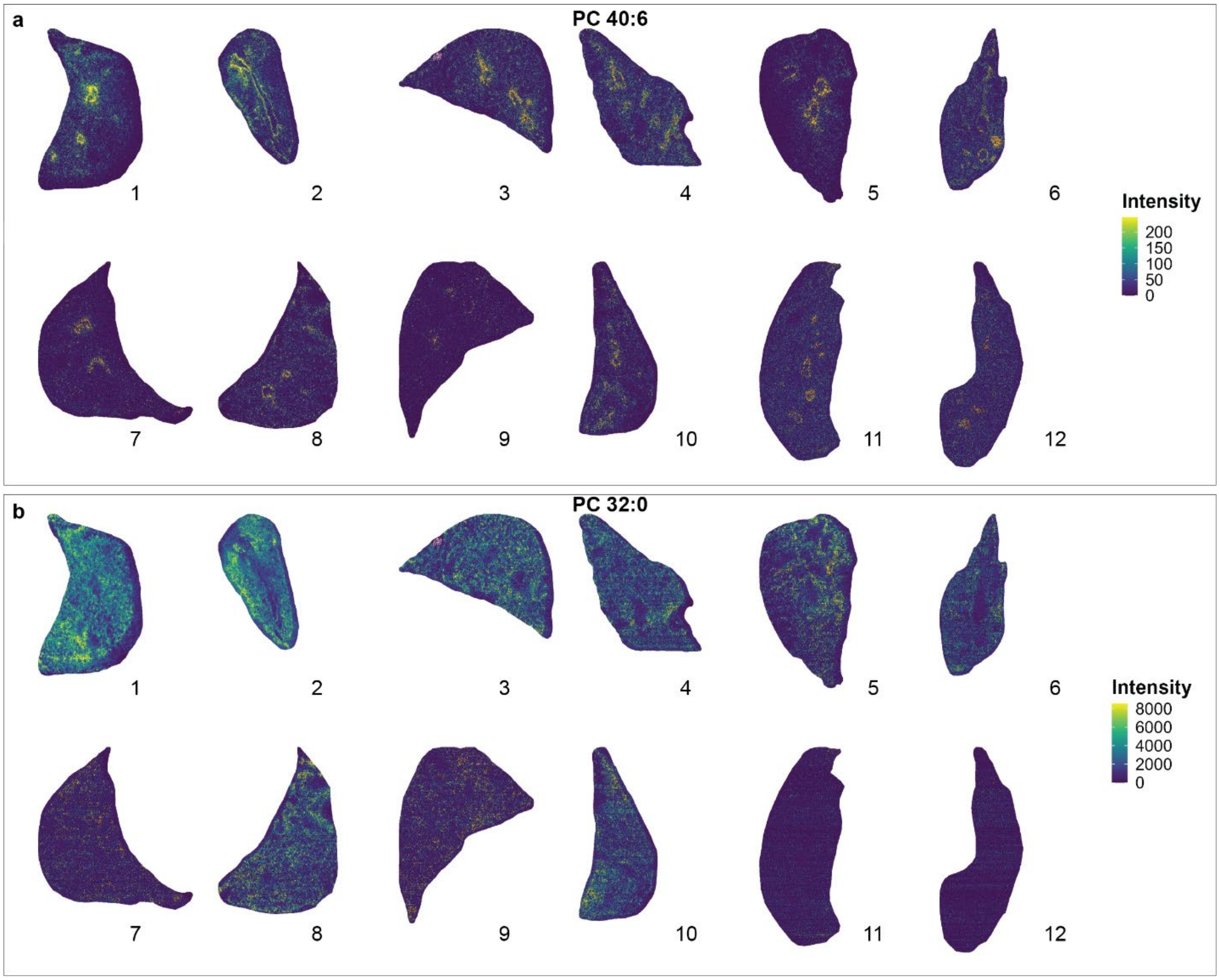

Supplementary Figure 5. Spatial distribution of individual phospholipid species identified in positive mode in sparse LOESS-normalized ion images. Ion images representing pixel intensities of annotated phospholipids in positive mode, including **a)** PC 40:6 and **b)** PC 32:0. Intensity distributions are included for each individual lung lobe acquired by positive mode and numbered in order of data acquisition.

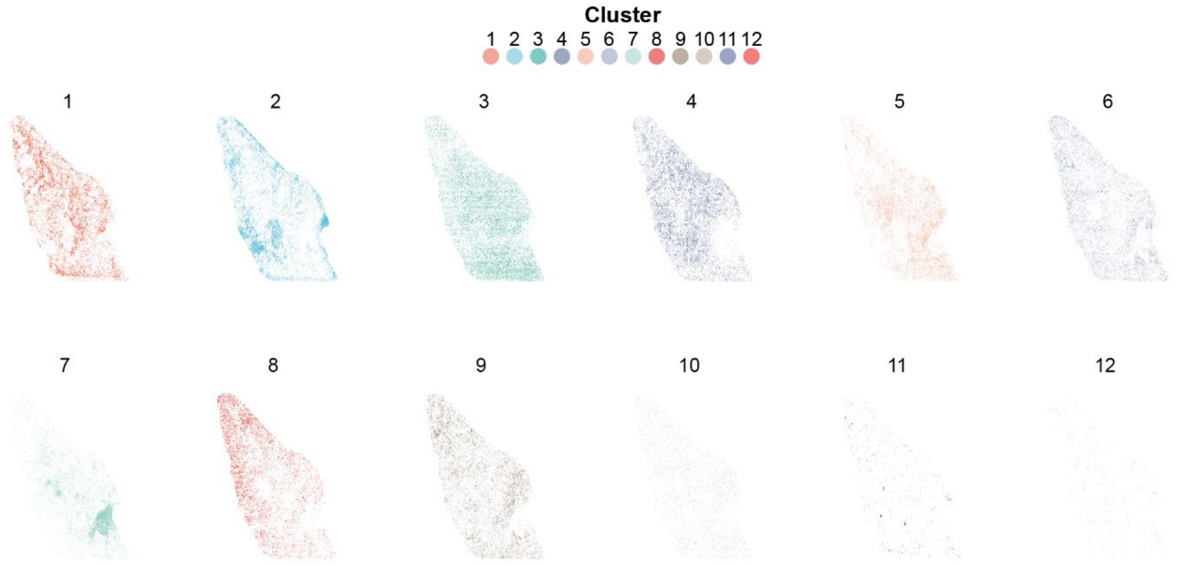

Supplementary Figure 6. Split cluster images depicting clusters corresponding to technical artifacts in positive mode. Images displaying grouped clusters based on unsupervised image segmentation. Clusters 3 and 11 represent technical artifacts related to matrix deposition and sample acquisition.

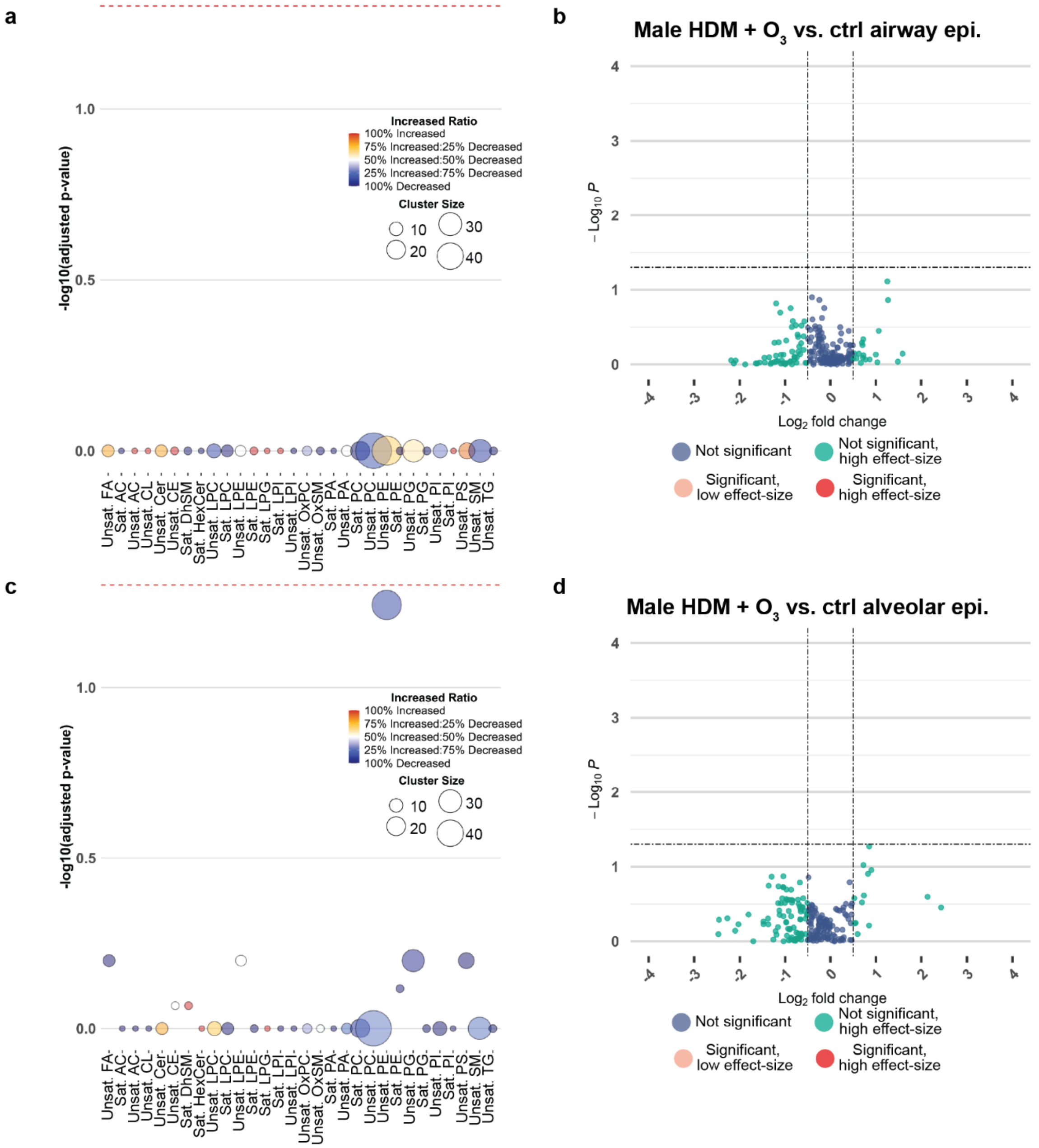

Supplementary Figure 7. Comparison of airway and alveolar epithelial changes in lipid composition between HDM + O_3 and control-treated male mice. a) Dot plot summarizing unsaturated (Unsat.), saturated (Sat.), or oxidized (Ox) lipid class enrichment results comparing male HDM + O_3 and control-treated airway epithelium. **b)** Volcano plot summarizing significantly altered lipids in the airway epithelium comparing the HDM + O_3 group relative to male control mice. **c)** Dot plot summarizing lipid class enrichment results comparing male HDM + O_3 and control-treated alveolar epithelium. **d)** Volcano plot summarizing significantly altered lipids in the alveolar epithelium comparing the HDM + O_3 group relative to male control mice. All dot plots and volcano plots used a p-value cutoff of $p < 0.05$ to determine statistical significance. The fold change direction for all panels is expressed as the abundance in the HDM + O_3 group relative to the control group. P-values for enrichment analyses were based on a Kolmogorov-Smirnov Test with FDR-correction. P-values for univariate analyses were determined based on a one-way ANOVA with Tukey's post-hoc analysis. A $\log_2$ fold-change of 0.5 (or a fold-change that is greater than 1) was used to define a high-effect size. Fatty acid (FA), acylcarnitine (AC), cardiolipin (CL), ceramide (Cer), cholesterol ester (CE), dihydrosphingomyelin (DhSM), glycosylceramide (HexCer), lysophosphatidylcholine (LPC), lysophosphatidylethanolamines (LPE), lysophosphatidylglycerol (LPG), lysophosphatidylinositol (LPI),

sphingomyelin (SM), phosphatidic acid (PA), phosphatidylcholine (PC), phosphatidylethanolamine (PE), phosphatidylglycerol (PG), phosphatidylinositol (PI), phosphatidylserine (PS), triacylglycerol (TG). Source data for panels a-d are provided as Source Data files.

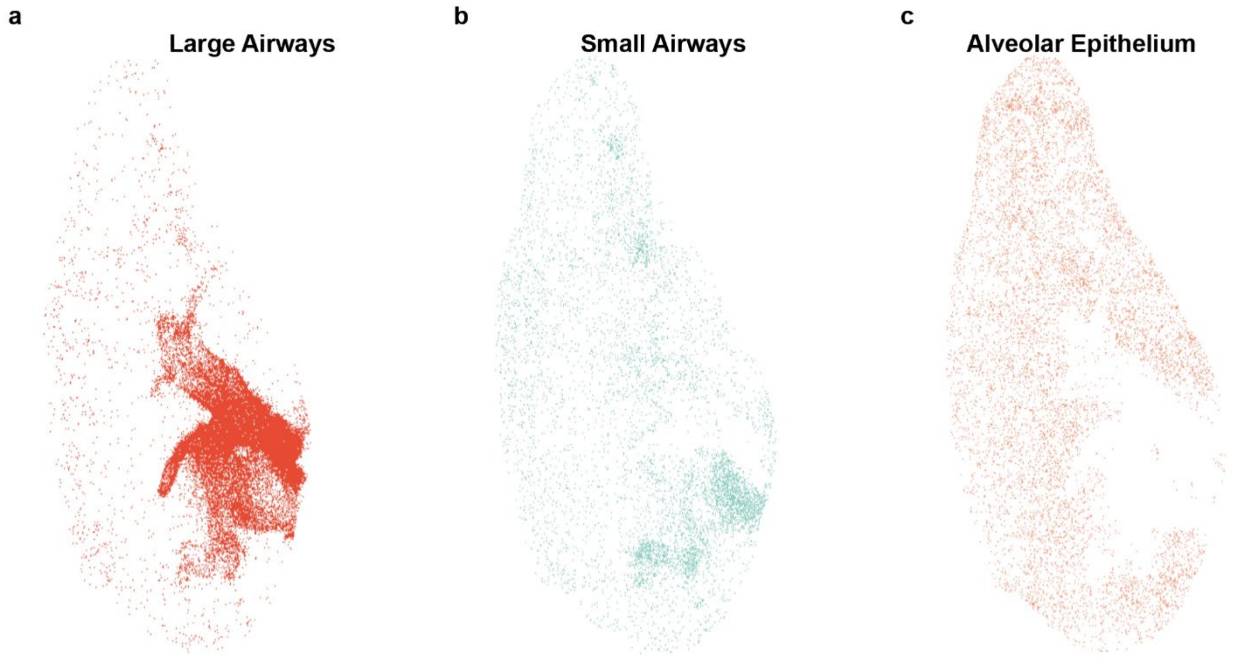

Supplementary Figure 8. Split cluster images distinguishing large airways, small airways, and alveoli. Images displaying grouped pixel clusters based on unsupervised image segmentation. **a)** large airways, **b)** small airways, and **c)** alveolar epithelium.

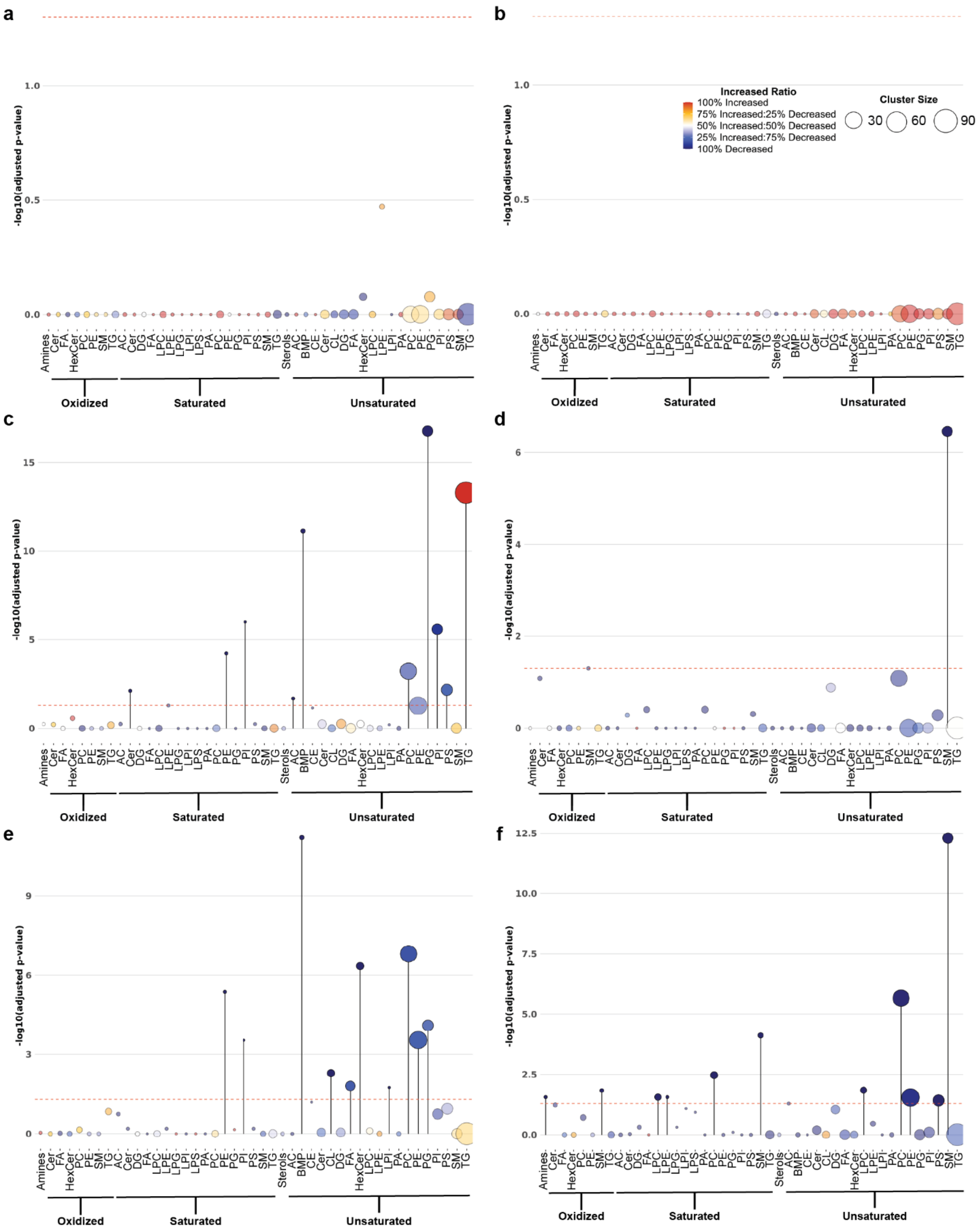

Supplementary Figure 9. Chemical similarity enrichment analysis of microdissected airways exposed to ozone or sensitized to HDM alone. Dot plots summarizing lipid class enrichment results comparing **a)** HDM-treated male vs. HDM female, **b)** ozone-exposed male vs. ozone-exposed female, **c)** control-treated male vs HDM-treated male, **d)** ozone-exposed male vs filtered air-exposed male, **e)** control-

treated female vs. HDM-treated female, and **f)** ozone-exposed female vs. filtered air-exposed female mice. All dot plots used a p-value cutoff of $p < 0.05$ to determine statistical significance. Enrichment analyses were based on a Kolmogorov-Smirnov Test with FDR-correction. P-values for univariate analyses were determined based on a one-way ANOVA with Tukey's post-hoc analysis in R using a 95% confidence interval and the default ANOVA function parameters in the base R library. The dotted lines on each panel represent FDR adjusted $p < 0.05$, and the proportion of lipids that are increased and decreased in each comparison are represented by red and blue shading, respectively. The number of lipids included in each class is indicated by the size of each point, and all comparisons were based on a sample size of 8 mice per treatment group. Fatty acid (FA), acylcarnitine (AC), cardiolipin (CL), ceramide (Cer), cholesterol ester (CE), dihydrosphingomyelin (DhSM), glycosylceramide (HexCer), lysophosphatidylcholine (LPC), lysophosphatidylethanolamines (LPE), lysophosphatidylglycerol (LPG), lysophosphatidylinositol (LPI), sphingomyelin (SM), phosphatidic acid (PA), phosphatidylcholine (PC), phosphatidylethanolamine (PE), phosphatidylglycerol (PG), phosphatidylinositol (PI), phosphatidylserine (PS), triacylglycerol (TG), diacylglycerol (DG), lysophosphatidylserine (LPS), bis(monoacylglycerol)phosphate (BMP). Source data for panels a-f are provided as Source Data files.
