# Supplementary material for: Resolving multi-image spatial lipidomic responses to inhaled toxicants by machine learning
Source: Nat Commun. 2025 Mar 26;16:2954. doi: 10.1038/s41467-025-58135-4 (PMC11947182; doi:10.1038/s41467-025-58135-4)
Supplement: Supplementary file 2 — Description of Additional Supplementary Files [file 41467_2025_58135_MOESM2_ESM.pdf]

Description of Additional Supplementary Files

File Name: Supplementary Data 1

Description: Combined annotation list for positive and negative ionization modes.

File Name: Supplementary Data 2

Description: Summary of lipid annotations by class, subclass, and saturation degree.

File Name: Supplementary Data 3

Description: Sample information for positive and negative ionization mode data acquisition.

File Name: Supplementary Data 4

Description: Top-5 lipids present in segmented airway and alveolar epithelial regions for each sample.

File Name: Supplementary Data 5

Description: Univariate statistical analysis results comparing lipid abundance in the HDM + O3 groups relative to control-treated mice based on a one-way ANOVA with Tukey's post-hoc analysis.
