# Supplementary material for: Resolving multi-image spatial lipidomic responses to inhaled toxicants by machine learning
Source: Nat Commun. 2025 Mar 26;16:2954. doi: 10.1038/s41467-025-58135-4 (PMC11947182; doi:10.1038/s41467-025-58135-4)
Supplement: Supplementary file 8 — Reporting Summary [file 41467_2025_58135_MOESM8_ESM.pdf]

nature portfolio

Corresponding author(s): Prof. Oliver Fiehn

Last updated by author(s): Feb 12, 2025

Reporting Summary

Nature Portfolio wishes to improve the reproducibility of the work that we publish. This form provides structure for consistency and transparency in reporting. For further information on Nature Portfolio policies, see our Editorial Policies and the Editorial Policy Checklist.

Statistics

For all statistical analyses, confirm that the following items are present in the figure legend, table legend, main text, or Methods section.

n/a	Confirmed	
☐	☒	The exact sample size (*n*) for each experimental group/condition, given as a discrete number and unit of measurement
☐	☒	A statement on whether measurements were taken from distinct samples or whether the same sample was measured repeatedly
☐	☒	The statistical test(s) used AND whether they are one- or two-sided<br>*Only common tests should be described solely by name; describe more complex techniques in the Methods section.*
☒	☐	A description of all covariates tested
☐	☒	A description of any assumptions or corrections, such as tests of normality and adjustment for multiple comparisons
☐	☒	A full description of the statistical parameters including central tendency (e.g. means) or other basic estimates (e.g. regression coefficient) AND variation (e.g. standard deviation) or associated estimates of uncertainty (e.g. confidence intervals)
☐	☒	For null hypothesis testing, the test statistic (e.g. *F*, *t*, *r*) with confidence intervals, effect sizes, degrees of freedom and *P* value noted<br>*Give P values as exact values whenever suitable.*
☒	☐	For Bayesian analysis, information on the choice of priors and Markov chain Monte Carlo settings
☒	☐	For hierarchical and complex designs, identification of the appropriate level for tests and full reporting of outcomes
☒	☐	Estimates of effect sizes (e.g. Cohen's *d*, Pearson's *r*), indicating how they were calculated

Our web collection on statistics for biologists contains articles on many of the points above.

Software and code

Policy information about availability of computer code

Data collection	Data processing of LC-MS/MS .RAW files was completed in MS-DIAL v4.70. Conversion of raw mass spectrometry imaging data files to .imzml format was completed using SCiLS Lab 2023c (Bruker Daltonics, Inc.).
Data analysis	All processing, analysis, and visualization of the resulting .imzml files was completed in R (v4.3.3, v4.4.1, and v4.4.2) using WSL2 or R Studio (v.2023.06.2+561) using RegioMSI (v1.0 and v1.1). RegioMSI (MIT license) was developed as part of this study and uses R packages available from either CRAN or Bioconductor. Data preprocessing in RegioMSI was completed using Cardinal (v.3.4.3 or v.3.6.2) and RegioMSI segmentation used Seurat (v.5.0.3 and v.5.1.0). All source code is available on Github at https://github.com/cschasestevens/RegioMSI and a compiled version of RegioMSI is available on Zenodo at https://zenodo.org/records/14834145 with the following DOI: 10.5281/zenodo.14834145.

For manuscripts utilizing custom algorithms or software that are central to the research but not yet described in published literature, software must be made available to editors and reviewers. We strongly encourage code deposition in a community repository (e.g. GitHub). See the Nature Portfolio guidelines for submitting code & software for further information.

Data

Policy information about availability of data

All manuscripts must include a data availability statement. This statement should provide the following information, where applicable:

- Accession codes, unique identifiers, or web links for publicly available datasets
- A description of any restrictions on data availability
- For clinical datasets or third party data, please ensure that the statement adheres to our policy

The raw and processed data generated in this study have been deposited in the Zenodo database under the accession code 10.5281/zenodo.14846221 [https://zenodo.org/uploads/14846221]. The data are available free of restrictions and can be downloaded directly from Zenodo. Source data for all graphs included as figure panels within this study have been deposited in the Zenodo database under the accession code 10.5281/zenodo.14846581 [https://zenodo.org/uploads/14846581]. Supplementary tables are included as Supplementary Data files with this manuscript. Unless otherwise stated, all data supporting the results of this study can be found in the article, supplementary, and source data files.

Research involving human participants, their data, or biological material

Policy information about studies with human participants or human data. See also policy information about sex, gender (identity/presentation), and sexual orientation and race, ethnicity and racism.

Reporting on sex and gender	This study did not involve participants, data, or biological material derived from human participants.
Reporting on race, ethnicity, or other socially relevant groupings	n/a
Population characteristics	n/a
Recruitment	n/a
Ethics oversight	n/a

Note that full information on the approval of the study protocol must also be provided in the manuscript.

Field-specific reporting

Please select the one below that is the best fit for your research. If you are not sure, read the appropriate sections before making your selection.

[x] Life sciences [ ] Behavioural & social sciences [ ] Ecological, evolutionary & environmental sciences

For a reference copy of the document with all sections, see nature.com/documents/nr-reporting-summary-flat.pdf

Life sciences study design

All studies must disclose on these points even when the disclosure is negative.

Sample size	All animal exposures and experiments were conducted following approved protocols reviewed by the UC Davis Institutional Animal Care and Use Committee in accordance with guidelines for animal research established by the National Institutes of Health. Left lung lobes from mice treated with either a combination of house dust mite and ozone or vehicle and filtered air were analyzed in this study (N=3 males and 3 females per group, total N=12). We did not perform sample-size calculation as we previously detected differences in lipid abundance using this treatment and exposure paradigm based on LC-MS/MS analysis (https://doi.org/10.1093/toxsci/kfac117). The results of our prior work revealed the greatest extent of lipidomic differences between the combined house dust mite and ozone-exposed and control mice. We determined that a smaller sample size including only these two groups would be sufficient for detecting statistically significant differences in our present study. Thus, we were able to circumvent common limitations of mass spectrometry imaging experiments regarding sample throughput and computational resources that prevent analysis of sample sizes typically incorporated in LC-MS/MS-based lipidomics studies.
Data exclusions	We excluded unknown peaks and manually-curated artifacts from all image segmentation and statistical analysis. These exclusions were made on the basis that technical artifacts derived from data acquisition would skew downstream analyses and image segmentation performance. Peaks were annotated based on LC-MS/MS from either the same tissue or from microdissected mouse lung tissue under identical experimental conditions. These filtering steps excluded technical artifacts and unknown peaks that likely correspond to in-source-fragments and adducts. However, our analysis package provides options to toggle these filtering steps for further exploration of unknown peaks.
Replication	We conducted this study to gain additional insights from our previous experimental protocol modeling combined allergen and air pollutant exposure in mice. The findings of this study validate previously reported results by us and other groups indicating lipidomic changes following combined house dust mite or ozone exposure. However, this study also expands upon these findings by determining the spatial localization of lipidomic changes, which is important for detailed mechanistic understanding of region-specific toxicants such as ozone. We did not directly replicate our mass spectrometry imaging experiment as the main focus this study was to provide a framework for mass spectrometry imaging analysis across biological replicates. We were also limited due to the time and resource intensive nature of preparing and analyzing samples using mass spectrometry imaging. Nonetheless, all aspects of this study could be replicated using our established experimental protocol and RegioMSI R package.

Randomization	Samples were randomized prior to data acquisition and processing to avoid technical bias. Mice were assigned to treatment groups according to cage number.
Blinding	Blinding was not possible as the same author was responsible for the conducting the exposure, data collection, and analysis of this study. Multiple practices were exercised to ensure all data were analyzed equally, including randomization during data acquisition and using identical code during analysis.

Reporting for specific materials, systems and methods

We require information from authors about some types of materials, experimental systems and methods used in many studies. Here, indicate whether each material, system or method listed is relevant to your study. If you are not sure if a list item applies to your research, read the appropriate section before selecting a response.

Materials & experimental systems

n/a	Involved in the study
☒	☐ Antibodies
☒	☐ Eukaryotic cell lines
☒	☐ Palaeontology and archaeology
☐	☒ Animals and other organisms
☒	☐ Clinical data
☒	☐ Dual use research of concern
☒	☐ Plants

Methods

n/a	Involved in the study
☒	☐ ChIP-seq
☒	☐ Flow cytometry
☒	☐ MRI-based neuroimaging

Animals and other research organisms

Policy information about studies involving animals; ARRIVE guidelines recommended for reporting animal research, and Sex and Gender in Research

Laboratory animals	BALB/c mice were purchased from Envigo, Inc. at 8-10 weeks of age for this study.
Wild animals	This study did not involve wild animals.
Reporting on sex	Both male and female mice were used in this study and were implemented in equal numbers within each treatment group. Information for each sample is included in Supplementary Material.
Field-collected samples	This study did not involve animals collected from the field.
Ethics oversight	Animal exposures and experiments were conducted following approved protocols reviewed by the UC Davis Institutional Animal Care and Use Committee in accordance with guidelines for animal research established by the National Institutes of Health.

Note that full information on the approval of the study protocol must also be provided in the manuscript.

Plants

Seed stocks	n/a
Novel plant genotypes	n/a
Authentication	n/a
